# High-Resolution Mapping of the Nordic Plastic Cycle Suggests Capacity Expansion for Both Mechanical and Chemical Recycling

**DOI:** 10.1021/acssusresmgt.5c00143

**Published:** 2025-10-03

**Authors:** Yunhu Gao, Xuewei Liu, Wu Chen, André Cabrera Serrenho, Ciprian Cimpan, Gang Liu

**Affiliations:** † Center for Low-Carbon Conversion Science & Engineering, Shanghai Advanced Research Institute, Chinese Academy of Sciences, Shanghai 201210, China; ‡ Department of Engineering, 2152 University of Cambridge, Trumpington Street, Cambridge CB2 1PZ, U.K.; § State Key Laboratory of Low Carbon Catalysis and Carbon Dioxide Utilization, Shanghai Advanced Research Institute, Chinese Academy of Sciences, Shanghai 201210, China; ∥ SDU Life Cycle Engineering, Department of Green Technology, 6174 University of Southern Denmark, 5230 Odense, Denmark; ⊥ College of Urban and Environmental Sciences, 12465 Peking University, Beijing 100871, China; # Institute of Carbon Neutrality, Peking University, Beijing 100871, China

**Keywords:** Material flow analysis, plastics, mechanical recycling, chemical recycling, pyrolysis, Nordic countries

## Abstract

Nordic countries are widely recognized for their leadership in sustainability initiatives and have implemented numerous projects to improve plastic waste recycling and utilization. However, the plastic consumption and waste management in these countries remain insufficiently understood due to the lack of dynamic, high-resolution plastic cycle maps that span the entire lifecycle. Here, we devised a polymer-level dynamic material flow analysis model by integrating data from disparate sources to simulate the historical cycle of 14 groups of polymers (1978–2020) and their recycling potentials by 2050 in five Nordic countries (Denmark, Finland, Norway, Sweden, and Iceland). The results show that the average per capita stock in Nordic countries in 2020 reached the saturation level (1100 kg per capita), which is the highest global value. Imported polymers far exceeded the domestic production. Most of the plastic waste was incinerated or landfilled, with the average recycling rate falling below 6%. Enhanced mechanical recycling could contribute to 27% of the regional demand, requiring 6.7 times the expansion of the current recycling capacity by 2050. The additional implementation of chemical recycling could potentially provide 22% of the regional demand, but the potential contribution of chemical recycling is compromised by the lack of industrial production in the region, implying the need for international collaborations. The results contribute to addressing key issues under discussion in the ongoing negotiations of the Intergovernmental Negotiating Committee for the Global Plastic Treaty.

## Graphical abstract

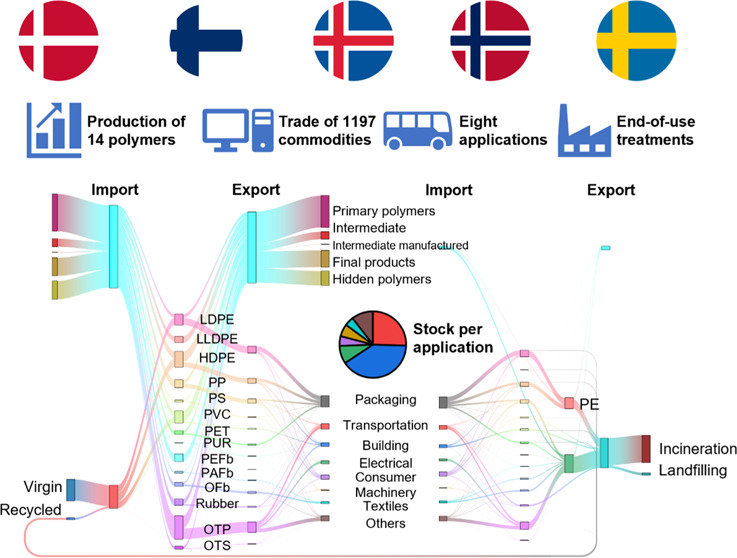

## 1 Introduction

Polymers, including plastics, fibers, and rubbers, are human-made materials. Plastic was first invented in the 1860s, developed for industry in the 1930s, and became one of the fastest-growing global industries in the 1950s.^1–3^ Polymer production has increased 230-fold since 1950, booming from 1.7 million tons per year to 400 million tons per year.^4,5^ About 350 million tons of plastic waste are generated per year globally,^6^ and 22% of them are dumped into the environment or openly burnt.^5^ Plastic litter has been documented in all environments, from the highest mountain (Everest)^7^ to the ocean deeps (the Mariana Trench),^8^ in rivers,^9^ and in the atmosphere.^10^ The plastic debris in the environment has persistent and detrimental impacts on the marine,^11,12^ terrestrial, and freshwater ecosystems,^13,14^ as well as wildlife^15^ and human well-being.^16^

The Nordic countries (Denmark, Finland, Norway, Sweden, and Iceland) are global leaders in environmental sustainability and climate actions but lag behind on circular economy performance, as shown by national “circularity gap” studies.^17^ The total plastic production of Nordic countries is much less than those of other large producers, but their per capita plastic consumption far exceeds that of other regions. For example, the per capita annual plastic consumption in Europe was 119.4 kg in 2022, while the per capita annual consumption in Denmark and Norway reached more than 200 kg.^18,19^ To support the achievement of United Nations Sustainable Development Goal 14, ‘Life Below Water’, which aims to conserve and sustainably use oceans, seas, and marine resources,^20^ the European Commission has introduced a range of policies and strategies focused on plastic waste management and recycling. A number of goals are proposed for 2030, for example, recycling 55% of plastic packaging waste by the amended Packaging and Packaging Waste Directive 1994,^21^ all packaging on the EU market being reusable or recyclable in an economically viable way by the New Circular Economy Action Plan and European Green Deal,^22,23^ and a 50% reduction of plastic litter in the sea and a 30% reduction in the release of microplastic into the environment by the Zero Pollution Action Plan.^24^ The policy landscape of the Nordics mainly follows EU directives and often has a high level of ambition.^25^ Over the past decade, the Nordics have initiated a series of projects to increase plastic recycling and reduce plastic pollution.^26–29^ The DRS for plastic bottles is the most successful plastic recycling scheme in the Nordic regions. DRS incentivizes the return of beverage containers by charging a deposit upon purchase and refunding it when the container is returned. The returned containers will be cleaned and reused, or the materials will be recycled to produce new products. More than 90% of PET bottles are returned through DRS in the Nordic countries.^30–32^

The DRS has greatly increased the recycling rate of PET bottles, but PET packaging only accounts for a small proportion of the total plastic consumption, and polymers with large consumption include polypropylene (PP), low-density polyethylene (LDPE), high-density polyethylene (HDPE), and polyvinyl chloride (PVC).^33^ The recycling rate of PVC in the Nordic region is estimated to be less than 13%, which is lower than the European average of 25%, and moreover, most PVC recycling seems to occur outside of the Nordic countries.^34^ Incineration remains the primary treatment method for plastic waste from households (excluding packaging), end-of-life (EoL) vehicles, and construction and demolition waste.^35^ In Sweden, only 10% of plastic waste was used for material recycling in 2020, while 87% of waste was incinerated for energy recovery.^36^ In Norway, 24% of plastic waste is collected for recycling, and only 9% of recyclate is used by industries.^37^ In addition, it is suggested that the current recycling rate in Europe may be overestimated because there is a substantial amount of plastic waste that has not been identified, known as ‘missing plastic’ (for instance, the share of plastics in municipal waste is difficult to obtain).^38^ Despite the rich policy framework, actual plastic waste management in the Nordic countries seems neither optimistic nor clear. In order to understand and promote the sustainability of plastic waste management in Nordic countries, there is an urgent need to obtain a complete picture of plastic cycles and recycling pathways.

Such a full picture of the plastic cycle requires a whole life-cycle perspective approach, which monitors the ‘upstream’ sources (e.g., raw material extraction, energy production, production and transportation of inputs) in order to reduce the impact of “downstream” processes (e.g., use phase, EoL treatment). Material flow analysis (MFA) is an effective method to quantify the plastic flows throughout ecological and socio-economic compartments. Previous studies have used MFA to study the plastic cycle, providing detailed polymer and product- and sector-specific assessments, including their flows to the environment.^33,39–41^ Dynamic MFAs have also been conducted to understand changes in plastic cycles over time and to develop future scenarios.^42–44^ However, most existing plastic MFAs of Europe analyze Europe as a whole and do not have a special resolution for the Nordic region or countries, except a few detailed analyses on individual countries, such as the United Kingdom,^41^ Switzerland,^45^ and Germany.^46^ Product- and sector-specific MFAs for plastics have only been conducted in Norway^37,47^ and Denmark.^48^ Norway is the only case with detailed import and export information compiled based on the HS code. However, such research including the comprehensive analysis of plastic-containing products trade remains limited, particularly for hidden polymers in products partially made of polymers.^49–51^

In this study, we aim to address these gaps by developing a comprehensive, polymer-level, high-resolution plastic MFA for Nordic countries. As one of the most developed regions globally and a region whose environmental profiles and policies have become a model for many to emulate, such a detailed analysis of the plastic cycle and EoL pathways in the Nordic countries can provide important reference points for developing region-specific plastic waste recycling and pollution control policies in other regions around the world.

## 2 Methods

We constructed a dynamic MFA model to display the flows of polymers from production to EoL processing in the Nordic countries, which is further used to envisage the necessary capacity of polymer recycling to support a sustainable future supply chain and waste management system in this region.

### 2.1 Historical Mass Flow of Polymers in the Five Nordic Countries

The inflows (to the use phase), stock-in-service (in-use phase), and waste generation (after the use phase) of plastic polymers in the five Nordic countries (namely, Denmark, Finland, Iceland, Norway, and Sweden) from 1978 to 2020 were quantified. The system boundary is shown in Figure S1 in the Supporting Information. We reconciled data from disparate sources, including production data of 14 groups of polymers: low-density polyethylene, LDPE; linear low-density polyethylene, LLDPE; high-density polyethylene, HDPE; polypropylene, PP; polystyrene, PS; polyvinyl chloride, PVC; polyethylene terephthalate, PET; polyurethane, PUR; PEFb, polyester fiber; PAFb, polyamide fibers, including nylon-6 and nylon-66; OFb, other fibers; rubbers, including styrene–butadiene rubber, butyl rubber, nitrile, and butadiene copolymer, polybutadiene, polyisoprene, polychloroprene, and acrylonitrile polybutadiene copolymer; OTP, other thermoplastics, including acrylonitrile butadiene styrene, expandable PS, styrene acrylonitrile resin, ethylene-vinyl acetate, polycarbonate, and poly­(methyl methacrylate); and other thermosets, OTS, including unsaturated polyester and epoxy resin. We considered their applications in eight sectors: packaging, transportation, building and construction, consumer and institutional products, electrical and electronic products, industrial machinery, textiles, and others, which have different lifetime distributions.^52^

The annual production data of polymers from 1978 to 2020 was obtained from the Independent Commodity Intelligence Service (ICIS) database.^53^ The amounts of traded polymers from 1990 to 2020 were estimated according to the Comtrade database.^54^ We considered 1197 products completely or partially made of polymers from the Harmonized Coding and Description System 2017 (HS 2017).^55^ The 1197 products were categorized into 5 groups, namely, 76 primary polymers, 68 intermediate forms of polymers, 115 intermediate manufactured plastic products, 107 final manufactured polymer products, and 831 hidden polymers. While the first four groups of polymers were identified by the United Nations Conference on Trade and Development,^49^ the 831 hidden polymer products partially made of polymers were identified in our previous study^56^ and used to comprehensively quantify the trade of polymers in Nordic countries for the first time.

More than 5.4 million trade record entries were accounted for from 1978 to 2020 in the model. To estimate the import and export of polymers in each product, we first inspected the mass of each trade flow by calculating the average price in USD/kg for each trade in different years. The top and bottom 2.5% prices were regarded as singular values and replaced by the average price of this product traded in the same year, and the weights of the trade flows with normal prices were used directly. Then, the corresponding trade weight with a singular price was estimated using the monetary value and the average price. It should be mentioned that some trade data in one year (e.g., export of all products in Denmark in 1997) or several years (export of three staple products listed in the Supporting Information from 1992 to 1997 in Sweden) were not reported. To solve these problems, the missing export data in Denmark in 1997 was estimated using the average exports of 1996 and 1998, and the exports of three staple products in Sweden from 1992 to 1997 were estimated by the import-to-export ratio for each product in 1998 and the annual import from 1992 to 1997.

Having obtained the corrected mass flow for trade in these countries, we then quantified the mass flow of embedded polymer p, weightP_p,i,im,y,r_, in imported product i to country c in year y, as eq 1, where the fraction of polymers among the total weight of product i, Tf_i_, and the fraction of polymer p among all polymers, *f*_p,i_, were extracted from our previous work.^56^ Then, the total amount of imported polymer p, import_p,c,y_, can be added up as shown in eq 2. A similar approach was executed for exported products before the consumption of polymer p in country c and year y, consumption_p,c,y_, was calculated as eq 3.

1 $${weightP}_{p,i,im,y,c}={weight}_{i,im,y,c}\times {Tf}_{i}\times f_{p,i}$$

2 $${import}_{p,c,y}=\sum_{i}{weightP}_{p,i,im,y,c}$$

3 $${consumption}_{p,c,y}={production}_{p,c,y+}{import}_{p,c,y}-{export}_{p,c,y}$$

where weight_i,im,y,c_ represents the amount of imported (im) product i in year y and country c; production_p,c,y_ stands for the production of polymer p in country c and year y; and export_p,c,y_ is the exported weight of polymer p in country c and year y.

The consumption of polymer p in year y and country c, consumption_p,c,y_, was allocated to different sectors, according to the typical distribution summarized from various sources in our previous work.^56^ Thus, the national annual input of polymers in sector a, country c, and year y, input2app_c,a,y_, was calculated as eq 4.

4 $${input2app}_{c,a,y}=\sum_{p}{conPoApp}_{p,a,c,y}$$

where conPoApp_r,a,c,y_ is the consumption of polymer p applied in sector a, year y, and country c.

Coupling the annual input of polymers to various sectors and lifetime distributions of products in various sectors, we could estimate the stock-in-service and waste generation in each sector, each year, and each country, as shown in eqs 5 and 6. The total amount of stock, applicationstock_c,a,n_, from sector a in year n and country c was quantified using eq 7. The total waste generation from polymer p, sector a, in year n and country c, applicationwaste_c,a,n,p_, was estimated using eq 8.

5 $${stock}_{c,a,y,n,p}={conPoAPP}_{p,a,c,y}\times {Sf}_{y,n,a}$$

6 $${waste}_{c,a,y,n,p}={conPoAPP}_{p,a,c,y}\times \left({Sf}_{y,n-1,a}-{Sf}_{y,n,a}\right)$$

7 $${applicationstock}_{c,a,n}=\sum_{y,p}{stock}_{c,a,y,n,p}$$

8 $${applicationwaste}_{c,a,n,p}=\sum_{y}{waste}_{c,a,y,n,p}$$

where Sf_y,n,a_ is the log–normal survival function of products in year n,^52^ which were put into use in sector a and year y.

To estimate the amount of traded waste polymers, we identified five waste streams from HS 2017,^55^ which are listed in Section 1 of the Supporting Information. Then, we used the same method described above to inspect and correct the traded weight of the waste polymers. The amount of recycled plastics was extracted from the ICIS database.^53^ Other waste polymers that were not recycled or exported were either landfilled or incinerated. The percentage of polymers sent to landfill or incineration was obtained from a number of literature sources (see Table S3 in Supporting Information for detailed data sources). As no data are available for Iceland, the average values of four Nordic countries are used.

### 2.2 Projected Demand, Stock-in-Service, and Waste in the Business-as-Usual (BAU) Scenario

We estimated the historical national per capita inflow and per capita stock in eight sectors (1978–2020) by dividing historical inflow and stock (from Section 2.1) by national populations.^57^ We used the per capita stock to quantify service provided in different sectors, except for the packaging sector (using per capita inflow instead) because of their very short lifetime (less than one year).

We further modeled the prospective demand by dynamic stock-driven and inflow-driven models depending on the patterns of historical per capita stock (Figure S3) and per capita inflow (Figure S4). Figure S3 shows that most per capita stock by sector fluctuated between 2010 and 2020, with five exceptions discussed below. For packaging materials, Figure S4 demonstrates that their inflow per capita has also stabilized across countries. These trends align with observations in the UK plastic case^58^ and other studies on the mass flow of bulk materials such as steel,^59,60^ where new inputs are primarily used to replace retired materials and meet population-driven demand. We assumed that the national packaging inflow per capita and the stock-in-service per capita for other sectors would saturate at different national levels (Figures S3 and S4).

The following sectoral stock per capita increased in the last decades, and the slope dropped: transportation in Norway and Sweden and building and construction in Denmark, Iceland, and Norway. We used the Gompertz function in eq 9 to fit a smooth transition. First, we assumed the stock per capita from 2047 to 2050 to be 110% of the stock per capita in 2020 according to the historical trend. Then, we fitted the curve using calculated sectoral stock per capita from 2016 to 2020 and 2047 to 2050, as shown in Figure S3.

9 $${applicationstock}_{c,a,n}=A\cdot \exp\left(-\exp\left(-B\left(year-C\right)\right)\right)$$

where *A*, *B*, and *C* are coefficients of the Gompertz function.

Coupling with the population forecast by the United Nations,^61^ we could calculate the national stock-in-service in each sector, as shown in Figure S5. Then, the demand for polymers in each sector, except for packaging, can be calculated by eq 10 and is displayed in Figure S6, reflecting both retired materials replacement and stock increment. For the packaging sector, we estimated the total demand for packaging polymers in each country by inflow per capita and population growth (Figure S6). The demand for all polymers in each sector has been estimated, which can be allocated to different polymers according to the average fractions between 2018 and 2020. Then, we could calculate the amount of waste using eq 6 as described above.

10 $${input2app}_{c,a,y+1}={applicationstock}_{c,a,y+1}-{applicationstock}_{c,a,y}+\sum_{p}{applicationwaste}_{c,a,y+1,p}$$

The demand for virgin polymers could be reduced by the recyclates supply. To estimate the theoretical contribution of recyclates in the business-as-usual (BAU) scenario, we first calculated the mechanical recycling rate of polymer p in year n and country c, RR_c,n,p_, using the recyclates (from ICIS) divided by generated waste, as shown in Table S4. In the BAU scenario, we assumed that national mechanical recycling rates of polymers are constant between 2021 and 2050 and used the average mechanical recycling rate between 2018 and 2020 to calculate the amount of recyclates of polymer p in sector a, year n (from 2021 to 2050), country c, and recycling round rd, recyclates_n,a,c,rd_ (eq 11).

11 $${recylates}_{p,n,a,c,rd}={applicationwaste}_{c,a,n,p,rd‐1}\times {RR}_{c,n,p}$$

### 2.3 Mechanical Recycling and Chemical Recycling Scenarios

Recycling polymers has become a key strategy to reduce the demand for virgin materials and minimize waste sent to incineration or landfilling, supported by various policies.^62^ Mechanical recycling and chemical recycling are two primary methods for recycling plastic waste, based on polymer characteristics and economic factors.

Mechanical recycling involves melting waste polymers at elevated temperatures and casting them into a new shape without affecting their molecular structure. This process is energy-efficient, cost-effective, and produces lower greenhouse gas emissions compared to chemical recycling.^63^ Consequently, mechanical recycling is prioritized wherever feasible. Chemical recycling includes the cleavage of the chemical bond of the polymer chain for the production of monomers and repolymerization. Chemical recycling can be further divided into two categories, selective chemical recycling and non-selective chemical recycling, depending on if original monomers can be obtained.^62^

Selective chemical recycling depolymerizes only five groups of polymers (PET, polyester fiber, polyamide fiber, PS, and PUR) into their original monomers for new polymer synthesis.

Non-selective chemical recycling uses thermochemical methods like pyrolysis and gasification to break down polymers into intermediate products such as pyrolysis oil.^64^ Pyrolysis is often preferred over gasification due to lower operating temperatures and greater availability of industrial infrastructure.^56,65^ Therefore, it is assumed in this study that pyrolysis rather than gasification is used. The collected waste polymers can be pyrolyzed, and the obtained pyrolysis oil will be sent to steam crackers to obtain monomers, e.g., ethylene, propylene, and other chemicals.

In order to reveal the potential contribution of different recycling technologies and quantify the potential recycling capability of Nordic countries in the future, we devised three recycling scenarios: (1) enhanced mechanical recycling scenario (R1), (2) chemical recycling with steam cracking scenario (R2), and (3) chemical recycling without steam cracking scenario (R3), as summarized in Table 1. The approaches and parameters in these three scenarios are the same as those in the BAU scenario unless specified.

**Table 1:** Assumptions of Various Scenarios Regarding the Future Nordic Plastic Cycle

scenarios	details
BAU scenario	•population growth forecasted by the UN
	•assumed saturated packaging inflow per capita and stock-in-service per capita in other sectors
	•only mechanical recycling
	•mechanical recycling rates for LDPE, LLDPE, HDPE, PP, PS, PVC, PET, and other thermoplastics remain at the current low value
enhanced mechanical recycling scenario (R1)	•build on the BAU scenario
	•only mechanical recycling
	•mechanically recycled rates for LDPE, LLDPE, HDPE, PP, PS, PVC, PET, and other thermoplastics will gradually reach the maximum possible recycling rates by 2035. For example, 61% of PP packaging waste is recycled^66^
chemical recycling without steam cracking scenario (R2)	•build on the R1 scenario
	•mechanical recycling and chemical recycling
	•selective chemical recycling technology for PS, PET, PUR, polyester fiber, and polyamide fiber and chemically converting them to their monomers for repolymerization
chemical recycling with steam cracking scenario (R3)	•build on the R2 scenario
	•mechanical recycling and chemical recycling
	•the collected waste polymers not used for selective chemical recycling are all processed by pyrolysis and steam cracking

The enhanced mechanical recycling scenario assumes that the highest realistic mechanical recycling rates for various polymers applied in different sectors, as summarized in Table S5 from the best available sources in our previous study,^56^ will be fully applied by 2035. Sectoral mechanical recycling rates are projected to increase linearly from 2025 levels to their maxima by 2035. Most recyclable plastics are assumed to be recycled once, except HDPE, which can be recycled twice due to its robust mechanical properties.^56^ PET packaging materials are assumed to be recycled into packaging in all scenarios rather than polyester fibers like other regions,^56^ as the region lacks polyester fiber production.

The chemical recycling without steam cracking scenario is built upon the enhanced mechanical recycling scenario by selectively depolymerizing five applicable polymers (PET, polystyrene (PS), PUR, polyester fiber, and polyamide fiber) and repolymerizing them to corresponding polymers. Polyester fiber, polyamide fiber, and PUR are not currently mechanically recycled. To estimate the maximum chemical recycling rates, we assumed that 80% of their wastes could be collected^67,68^ and 80% of collected wastes could be sorted out for chemical recycling.^56^ Considering the technical routes of chemical recycling, 90%, 90%, and 80% yields were assumed for polyester fiber, polyamide fiber, and PUR in the chemical plants, respectively.^56^ In contrast with polyester fiber, polyamide fiber, and PUR, PET and PS can be mechanically recycled once in the model. In the first round, 80% collection rates were assumed, and the difference between the 80% collection rate and the sectoral mechanical recycling rate is called sorting and recycling loss. Half of the sorting and recycling loss was assumed suitable for chemical recycling. PET and PS were assumed to be not suitable for mechanical recycling in the second round. Thus, a collection rate of 80% and a sorting rate of 80% were assumed for the chemical recycling process. Yields of 90% and 80% in chemical recycling plants were assumed for PET and PS, respectively.^56^ The maximum chemical recycling rates were assumed to be implemented from 2045 to 2050, and the chemical recycling rates were assumed to linearly increase from 0 in 2035 to 2045.

The chemical recycling with steam cracking scenario focuses on processing polymers that cannot be selectively depolymerized into their corresponding monomers. It complements the chemical recycling without steam cracking scenario by utilizing pyrolysis and steam cracking to recycle all remaining polymer waste unsuitable for mechanical recycling or selective depolymerization. All collected waste polymers not processed by other methods are assumed to undergo pyrolysis to produce pyrolysis oil, which is then hydrogenated and fed into steam crackers. The steam crackers convert the pyrolysis oil into valuable monomers, such as ethylene, propylene, benzene, toluene, xylene, and butadiene, which are used for synthesizing virgin polymers. A collection rate of 80% was assumed for all of the polymers. Figure S2 in the Supporting Information exemplifies the mass flow in chemical recycling with steam cracking using LDPE used as packaging materials.

It was assumed that 72.5% of polymer waste processed through pyrolysis is converted into pyrolysis oil, except for PVC, and the remaining 27.5% converted into gas and char.^56^ For PVC, which releases hydrogen chloride (HCl) during heating, a 58% HCl weight fraction was deducted from the total weight, with the remainder being processed as feedstock for pyrolysis. The yields of ethylene, propylene, and other products from pyrolysis oil in the steam cracker were assumed to be the same as naphtha,^56,69^ with ethylene and propylene fractions of 0.324 and 0.168, respectively. More details on the yield and utilization of other chemicals can be found in our previous study.^56^

## 3 Results

### 3.1 The High-Resolution Plastic Cycle of the Nordic Countries in 2020

Figure 1 shows the national mass flow of polymers in Denmark, Finland, Norway, and Sweden in 2020. The mass flows in Iceland were negligible compared with the other four countries, and its results are shown in Supporting Information. Imports of products were higher than domestic production in all five countries. Sweden had the highest production (765 kt a^–1^) and highest export (2300 kt a^–1^) among all countries. Denmark produced negligible virgin polymers, and its exports were almost equal to domestic consumption. Norway had the highest domestic polymer consumption (1300 kt a^–1^) and stock (11.4 Mt) in 2020. Figures 1 and 2d indicate that packaging consumed more polymers than all other sectors in all of the countries.

**Figure 1:**
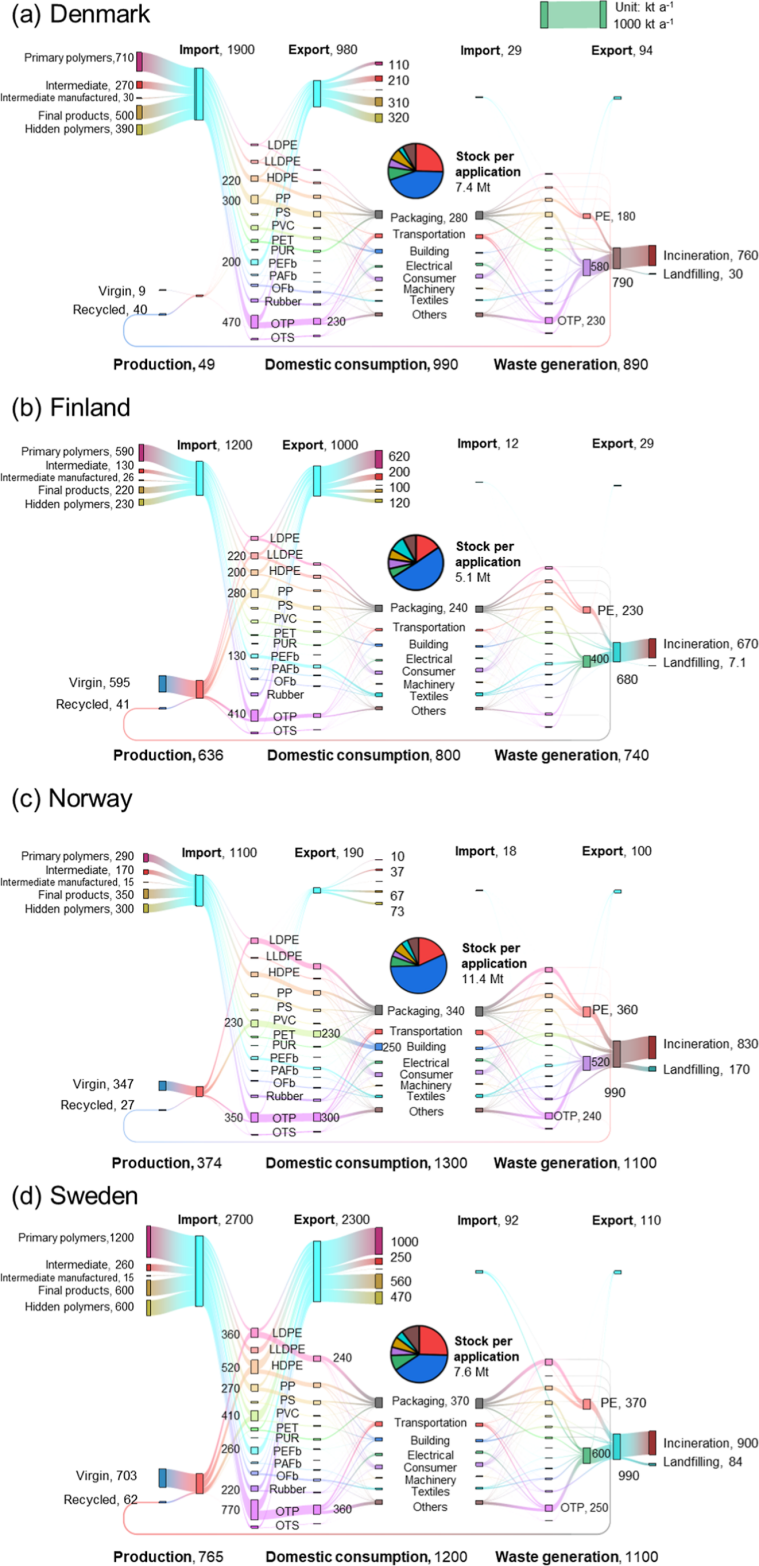
The year 2020 polymer-level plastic cycle in (a) Denmark, (b) Finland, (c) Norway, and (d) Sweden. The pie charts represent the amount of stock in service in various applications. The thickness of the line stands for the mass flow of polymers in kilotons per annum (kt a^–1^). List of polymers: low-density polyethylene, LDPE; linear low-density polyethylene, LLDPE; high-density polyethylene, HDPE; polypropylene, PP; polystyrene, PS; polyvinyl chloride, PVC; polyethylene terephthalate, PET; polyurethane, PUR; PEFb, polyester fiber; PAFb, polyamide fiber; OFb, other fibers; OTP, other thermoplastics; OTS, other thermosets. Building: building and construction, electrical: electrical and electronic products, consumer: consumer and institutional products, and machinery: industrial machinery.

**Figure 2:**
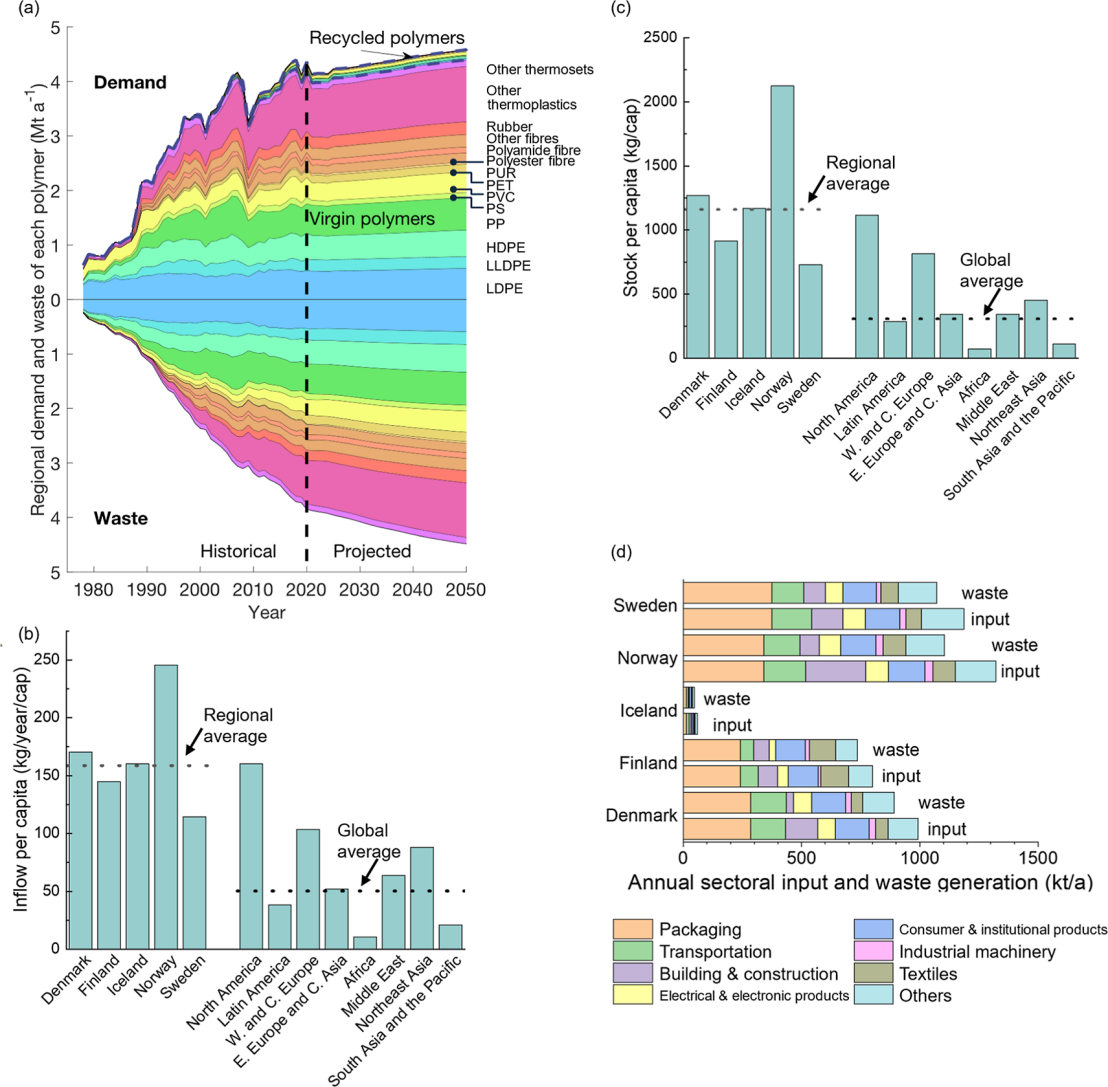
Regional annual demand for polymers and waste generation in the five Nordic countries. (a) Historical and projected regional annual demand per polymer. (b) The annual inflow per capita in the five Nordic countries in 2020 compared with other regions of the world.^56^ W. and C. Europe stands for Western and Central Europe, and E. Europe and C. Asia is Eastern Europe and Central Asia, respectively. (c) The stock-in-service per capita in the five Nordic countries in 2020 compared with other regions of the world.^56^ (d) Annual input per sector in each country in 2020.

Polymer wastes from four countries amounted to 890, 740, 1100, and 1100 kt a^–1^, respectively, in 2020. Packaging has the highest fraction of waste, accounting for around one-third of waste generation in all countries. Less than 13% of waste was exported to other countries, and most domestically processed waste was incinerated (higher than 70% in all five countries) rather than landfilled. Only less than 6% of polymer waste was recycled for material production domestically in these five countries. The non-recycled polymer waste reached 3.7 Mt a^–1^ in the Nordic region in 2020. The mass flow has been validated by a national study in Denmark^48^ in 2016, and detailed comparisons can be found in Section 2 of the Supporting Information.

### 3.2 Historical and Future Plastic Demand and Waste Generation

Figures 2b and 2c display the historical total annual per capita inflow and per capita stock in Nordic countries in comparison with eight regions in the world. The average per capita inflow and stock in Nordic countries are close to the highest global values (160 kg/cap/year and 1100 kg/cap, respectively, in North America in 2020).^56^ Norway has the highest national input flow, stock-in-service, and waste generation per capita among all Nordic countries, while Sweden has the lowest input flow, stock, and waste per capita.

Figure 2a shows that the demand for polymers will grow to 4.6 Mt a^–1^ by 2050, and not-recycled waste will increase to 4.5 Mt a^–1^ in the BAU scenario. Other thermoplastics, LDPE, PP, PVC, HDPE, and LLDPE together, account for 74% by 2050. These polymers are mainly used in packaging (LDPE, PP, HDPE, and LLDPE), building and construction (PVC), transportation, electrical and electronic products, and others (other thermoplastics).^56^ The deposit-refund system (DRS) in four Nordic countries has been highly successful, achieving return rates of 85%–95% by 2022. However, the system targets only PET beverage bottles, which account for less than 10% of the total polymer demand (Figure 2a). PET beverage bottles represent an even smaller share of this demand. Consequently, the high recycling rates achieved through the DRS currently have a limited impact on the overall polymer recycling rate. If the current low plastic recycling rates are kept, the contribution of recyclates will be only 4% by 2050, which needs to be improved to increase resource efficiency and decrease the dependency on fossil fuels. The high resolution of our model enabled the allocation of demand, stock-in-service, and waste in the BAU scenario to various polymers, countries, and applications (Figure S8).

### 3.3 Potential Impact of Polymer Recycling

To reduce the demand for virgin polymers and the corresponding fossil fuel extraction, it is necessary to recycle more waste polymers through mechanical and chemical recycling technologies. Herein, we devised three scenarios to reveal the potential contribution of three recycling technologies and, thus, to support policymaking.

Figure 3 displays the potential impacts of various mitigation options on virgin polymer demand and nonrecycled waste to be landfilled or incinerated. The allocation of demand, stock-in-service, and waste generation to polymers, sectors, and countries in different scenarios can be found in Figures S9–S11 in the Supporting Information.

**Figure 3:**
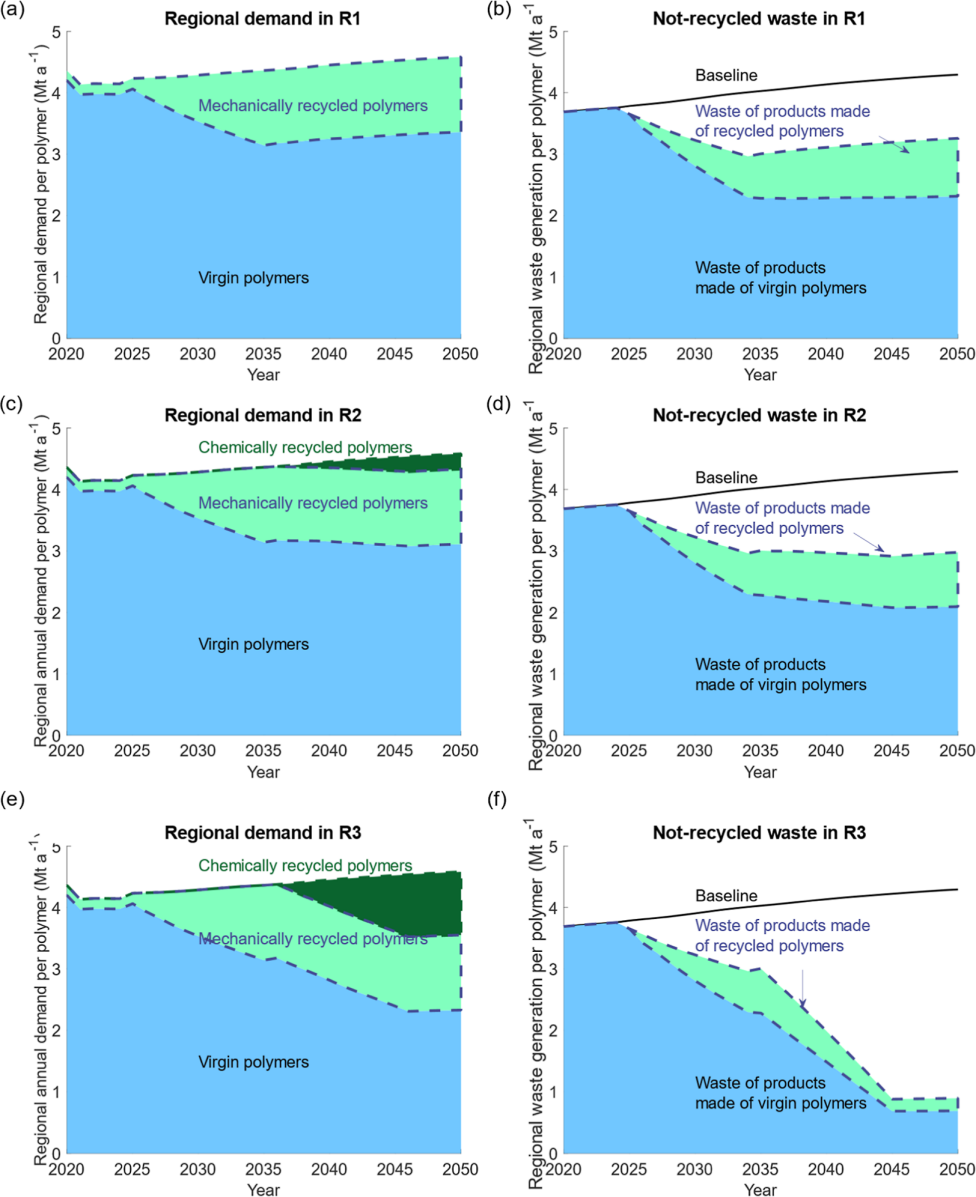
Potential impacts of various mitigation interventions. (a) The supply of various polymers and (b) not-recycled polymer wastes in the enhanced mechanical recycling scenario. (c) The supply of polymers and (d) not-recycled wastes in the chemical recycling without steam cracking scenario. (e) The supply of polymers and (f) not-recycled wastes in the chemical recycling with steam cracking scenario.

Figures 3a and 3b show that full implementation of enhanced mechanical recycling could contribute to 27% of the regional demand by 2050, and the amount of nonrecycled waste will drop by 24% compared with the BAU scenario. The highest recycling rate of 27% was comparable with Klotz et al.’s study on Switzerland.^66^ Compared with 0.17 Mt a^–1^ recyclates in 2020 (0.19 Mt a^–1^ recyclates in 2050) in the BAU scenario, the recycling capacity needs to increase 6.6 times from the current levels. However, it is not easy to actualize the application of recyclates. Identifying the proper applications of recyclates, advanced sorting technologies, and regulations is necessary.^67,68^ Specifically, 29% of nonrecycled waste is expected to come from polymers used more than once by 2050 (Figure 3b).

Figure 3c shows that chemical recycling without steam cracking could potentially contribute to only 5% of the regional demand for polymers. Applying chemical recycling is applied without steam cracking, the amount of nonrecycled waste will drop to 70% of the BAU (Figure 3d). Polyester fiber and polyamide fiber have the highest chemical recycling potentials. However, the production of PET, PUR, PS, polyester fiber, and polyamide fiber is negligible in the Nordic countries (Figure 1), implying the shortage of engineering capability to implement chemical recycling of these polymers. Exporting collected corresponding waste to other countries with relevant capacity might be an option, e.g., to Germany, as seen in the current practice.

The potential contribution of chemical recycling can be theoretically expanded to 22% of the regional demand for polymers in 2050 (Figure 3e) by introducing pyrolysis of polymer waste not suitable for selective depolymerization in the previous scenario for pyrolysis oil, which is later blended with naphtha and fed into steam crackers to produce ethylene and other raw materials.^69^ Polyethylene and PP are two important recyclates from this technology route. The amount of nonrecycled waste will be reduced to 21% of the BAU level, as shown in Figure 3f. Local pyrolysis plants^64^ and polyethylene and PP production capability (Figure 1) suggest that this is a technically viable solution. But the reported current capacity^64^ of 16 kt a^–1^ should be seen as a demonstration, and international collaboration should be combined with local capacity expansion to realize the potential contribution of chemical recycling. The related greenhouse gas emissions and cost should be further investigated to justify its development. The potential contribution of mechanical and chemical recycling is the highest in Norway and Sweden, as shown in Figure S11, due to their high national consumptions.

### 3.4 Discussion

We provided a detailed polymer-level characterization of the regional mass flow of polymers in the Nordic countries in this analysis. Their polymer supply heavily depends on import, and EoL waste is mostly incinerated. Urgent changes are needed to realize a sustainable regional polymer supply chain and waste management system. Enhanced mechanical recycling could contribute to 27% of the regional demand by 2050. However, improving the mechanical recycling rate requires collective action from all stakeholders of the supply chain. Design for recycling, identifying suitable applications for recyclates, customer’s awareness and preference, and policy incentives are key factors.

#### 3.4.1 Trade-Offs in Recycling Method Selection

Combining chemical recycling, recyclates potentially contribute to half of the regional demand. However, lack of regional production of applicable polymers (Figure 1) limits the potential of local selective chemical recycling, which may require international collaboration in the future. Although chemical recycling offers potential environmental benefits such as reducing fossil fuel consumption and greenhouse gas emissions, its economic viability for commercial-scale operations has yet to be demonstrated.

Chemical recycling is used if waste polymers degrade severely for mechanical recycling or if virgin-like quality of the recyclate polymer is needed. Although the energy consumed by the chemical process is generally higher than for mechanical recycling,^70^ chemical recycling is an invaluable method to use plastic waste as a raw material source and achieve a zero plastic waste goal, especially for waste streams that were previously energetically valorized by combustion. So far, chemical recycling has been confined to demonstration plants and smaller factories. The commercial success of chemical recycling depends largely on the costs and availability of selected waste streams and also on political regulations that make recycling processes more attractive. While the commercial success of polyamide fiber chemical recycling is well-documented due to the high cost of its virgin counterpart, the economic performance of other selective recycling technologies, e.g., PET and PS recycling, remains unclear.^70^ Local industrial practice has demonstrated the capability of recycling waste polymers by pyrolysis and steam cracking, which requires scaling up. However, the associated high energy cost is a potential hurdle for the large-scale commercial operation.^70^

Mechanical recycling, the only commercial-scale form today, incurs operating process costs of $0.05–$0.2 per kg of polymer waste, while chemical recycling costs $0.3–$1 per kg.^71^ The costs of mechanical recycling in Western Europe and North America are higher than those in other regions. In Western Europe, the profits of recycling PP, PET, and PVC are lower than $0.5 per kg of polymer, while the profits of recycling PS could reach $1–1.5 per kg.^72^

The prices range from roughly $1 to 2 per kg for commodity polymers up to tens of dollars per kg for specialty plastics.^73^ For commodity plastics, pyrolysis or gasification is claimed to be superior to mechanical recycling due to the extensive sorting efforts required.^74^ If commodity polymer waste is too mixed or contaminated, then the sorting process becomes labor-intensive and costly, even exceeding the cost of producing virgin polymers. However, the effectiveness of chemical recycling and mechanical recycling depends heavily on factors such as the quality of the incoming material stream, the quality standards of the recycled product, and the energy offsets associated with recovery processes. For engineering and high-performance plastics, efficient sorting is highly advantageous, as it significantly reduces the environmental impact of polymer waste by enabling the use of mechanical recycling or depolymerization technologies to produce recycled polymers suitable for applications similar to those of virgin materials. Among chemical recycling technologies, pyrolysis offers high feasibility and product yield with lower requirements for waste sorting. Catalytic cracking can yield high-purity, value-added products but relies on costly catalysts. Hydrocracking produces highly saturated liquid products of greater value without the need for further processing; however, its high capital and operating costs, along with complex chemical and catalyst requirements, limit its feasibility for large-scale production.^65^

Leveraging multisource high-resolution data, we developed high-precision plastic flow models for representative regions. The quantification and comparison of plastic cycles across the Nordic countries reflect regional differences. We assessed the impacts of various recycling technologies to provide insights into future recycling strategies. These results contribute to addressing key issues under discussion in the ongoing negotiations of the Intergovernmental Negotiating Committee for the Global Plastic Treaty.

#### 3.4.2 Uncertainty Analysis and Sensitivity Analysis

The present study reconciled data from the best available high-resolution sources, e.g., production data by the ICIS database and trade data by the Comtrade database, and accounts for a comprehensive list of polymer product trade including hidden polymers to depict the mass flow of polymers in the Nordic countries. The ICIS database was used to extract the complete set of recycled polymers. ICIS provides data on annual plastic production derived from recycled materials. This production-based data is generally more accurate and reliable than the estimates based on the collection and recycling rate of plastic waste used in some studies.^19,75^ Trade volumes in terms of weight are not always reported for all HS codes in the UN Comtrade database, but trade values are consistently available. We estimated the missing trade volumes using trade value data and average prices when the weight was missing or when the price was too high or too low. As mentioned above, the case study of Norway utilized HS codes to identify trade quantities for plastic products, with highly detailed codes.^47^ However, other countries lack such comprehensive data. This study used the Comtrade database of the United Nations and considered 831 hidden polymers embedded in products partially made of polymers.

The imported weight of polymer p, embedded in product i, year y, and region r, was calculated by eq S1, where the composition of polymers was estimated based on databases (e.g., Ecoinvent^76^ and Valpak^77^), commercial websites (e.g., Alibaba and Amazon), and the literature. The composition of polymers in primary polymers, intermediate polymer forms, and intermediate manufactured plastic products has lower uncertainty as their polymer content is well-defined and specific. For example, HS code 390210 refers to PP in primary forms, indicating a 100% PP fraction. The estimation of polymer fractions in the final manufactured polymer products and hidden polymers introduces uncertainty. To mitigate this, we refer to more than one data source for each product code. However, few studies have accounted for hidden polymers in trade flows, limiting the opportunities to compare our estimates with those of existing research. Drewniok et al. have estimated polymer fractions of over 400 PRODCOM codes,^58^ and our estimates are generally consistent with theirs.

The results are also subject to uncertainties arising from the assumptions. To demonstrate the impact of the parameters, we arbitrarily increased and decreased the maximum recycling rates by 20%, respectively. Consequently, the amount of recycled polymers in 2050 increased 14% and decreased 15%, respectively, and the amount of nonrecycled waste decreased 5% and increased 6%, respectively. The details of the sensitivity analysis are shown in Table S7.

In the future, we advocate for the adoption of similarly structured data in the Nordic region and beyond. However, the study is still subject to some limitations due to data availability, unpredictable future technology development, and policy. The variations of polymer distribution in various sectors may exist in the Nordic countries. Advances in chemical recycling technologies could potentially substantially increase the yield of recycling. While the selectivity of pyrolysis oil to desired ethylene and propylene is currently relatively low, the direct catalytic conversion of waste polymers to ethylene and propylene is being extensively investigated.^78^ Therefore, this study could only reflect our best estimation based on the currently available technology.

## Supplementary Material



## Notes

Details of the model and results (PDF)

## Abbreviations

LDPE: low-density polyethylene
LLDPE: linear low-density polyethylene
HDPE: high-density polyethylene
PP: polypropylene
PS: polystyrene
PVC: polyvinyl chloride
PET: polyethylene terephthalate
PUR: polyurethane
PEFb: polyester fiber
PAFb: polyamide fiber
OFb: other fibers
OTP: other thermoplastics
OTS: other thermosets
