# Supplementary material for: High-Resolution Mapping of the Nordic Plastic Cycle Suggests Capacity Expansion for Both Mechanical and Chemical Recycling
Source: ACS Sustain Resour Manag. 2025 Oct 3;2(11):2024–34. doi: 10.1021/acssusresmgt.5c00143 (PMC12676422; doi:10.1021/acssusresmgt.5c00143)
Supplement: Supplementary file 1 [file rm5c00143_si_001.pdf]

Supporting Information for ss

# High-resolution mapping of the Nordic plastic cycle suggests capacity expansion for both mechanical and chemical recycling

*Yunhu Gao[1,2,3], Xuewei, Liu[4], Wu Chen[4,*], André Cabrera Serrenho[2,*], Ciprian Cimpan[4], Gang Liu[5,6]*

[1]*Center for Low-Carbon Conversion Science & Engineering, Shanghai Advanced Research Institute, Chinese Academy of Sciences, Shanghai, 201210, China*

[2]*Department of Engineering, University of Cambridge, Trumpington Street, Cambridge, CB2 1PZ, United Kingdom*

[3]*State Key Laboratory of Low Carbon Catalysis and Carbon Dioxide Utilization, Shanghai Advanced Research Institute, Chinese Academy of Sciences, Shanghai, 201210, China*

[4]*SDU Life Cycle Engineering, Department of Green Technology, University of Southern Denmark, 5230 Odense, Denmark*

[5]*College of Urban and Environmental Sciences, Peking University, Beijing 100871, China*

[6]*Institute of Carbon Neutrality, Peking University, Beijing 100871, China*

**Corresponding author. E-mail address: wuc@igt.sdu.dk, ag806@cam.ac.uk*

## 1. System boundary, trade data and other model parameters

Figure S1 shows the system boundary of the model for each Nordic country.

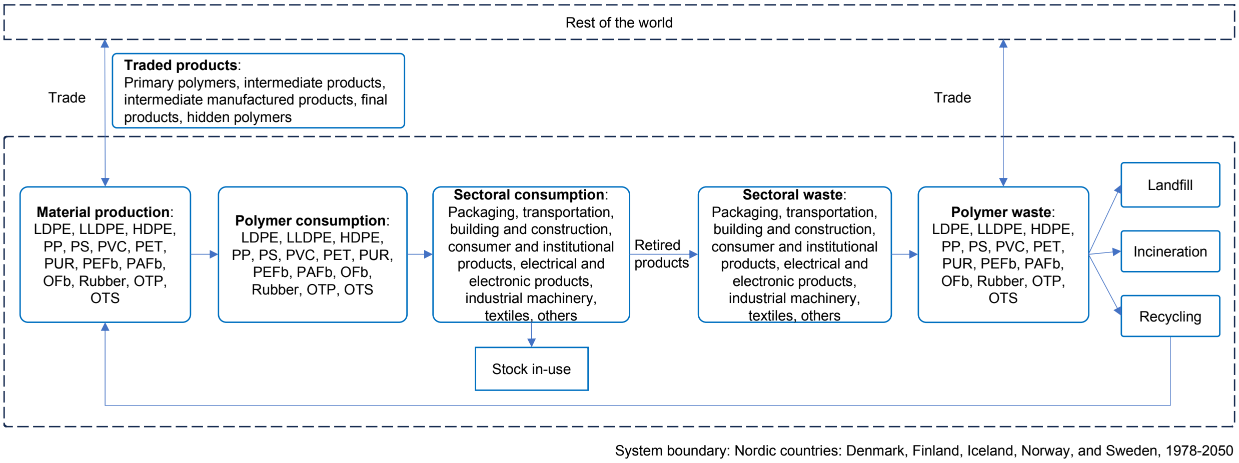


**Figure S1.** System boundary for the analysis on the mass flow of polymers in Nordic countries.

The export of commodities listed in Table S1 was not reported by Sweden from year 1992 to 1998. The export of all commodities in 1997 was not reported by Denmark. The missing data has been estimated as descripted in Methods.

**Table S1.** List of commodities whose export were not reported in Sweden between 1992 and 1997

| Code | Description |
|---|---|
| 390110 | Polyethylene - specific gravity <0.94 in primary forms |
| 390120 | Polyethylene - specific gravity >0.94 in primary forms |
| 390190 | Ethylene polymers; in primary forms, n.e.c. in heading no. 3901 |

Table S2 lists the traded polymer waste in Comtrade database.

**Table S2.** List of traded polymer waste

| Code | Description |
|---|---|
| 391510 | Waste, parings and scrap, of plastics - Of polymers of ethylene |
| 391520 | Waste, parings and scrap, of plastics - Of polymers of styrene |
| 391530 | Waste, parings and scrap, of plastics - Of polymers of vinyl chloride |
| 391590 | Waste, parings and scrap, of plastics - Of other plastics |
| 400400 | Waste, parings and scrap of rubber (other than hard rubber) and powders and granules obtained therefrom |

Table S3 displays the data sources of the fractions of end-of-use polymer waste treatment methods.

**Table S3.** Data source of treatment of polymers in four Nordic countries

| Country | Data source |
|---|---|
| Denmark | 1–4 |
| Finland | 1, 5–10 |
| Iceland | Average value of the other four countries |
| Norway | 9 |
| Sweden | 1, 11–17 |

Table S4 shows the average mechanical recycling rates of various polymers in each country between 2018 and 2020.

**Table S4.** Average mechanical recycling rates of various polymers between 2018 and 2020

| Polymers | Denmark | Finland | Iceland | Norway | Sweden |
|---|---|---|---|---|---|
| LDPE | 11.8% | 5.4% | NA* | 1.6% | 4.0% |
| LLDPE | 7.0% | 9.1% | NA | 2.5% | NA |
| HDPE | 6.7% | 10.6% | NA | 4.2% | 7.5% |
| PP | 2.1% | 2.1% | NA | 4.8% | 3.0% |
| PS | 8.3% | 8.0% | NA | 7.6% | 9.3% |
| PVC | 25.2% | 23.3% | NA | 7.0% | 21.6% |
| PET | 14.6% | 17.0% | NA | NA | 20.5% |
| PUR | NA | NA | NA | NA | NA |
| Polyester Fiber | NA | NA | NA | NA | NA |
| Polyamide Fiber | NA | NA | NA | NA | NA |
| Other Fiber | NA | NA | NA | NA | NA |
| Rubber | NA | NA | NA | NA | NA |
| Other thermoplastics | 0.9% | 4.8% | NA | 1.3% | 0.8% |
| Other thermosets | NA | NA | NA | NA | NA |

*NA: Not applicable

Table S5 shows the highest realistic recycling rate of polymers applied in different sectors

**Table S5.** Highest realistic recycling rate of polymers applied in different sectors[18]

| Applications / Polymers | Packaging[19] | Transportation[19] | Building & construction[19] | Electrical & electronic products[20] | Consumer & institutional products* | Industrial machinery# | Textiles[21,$] | Other[22] |
|---|---|---|---|---|---|---|---|---|
| LDPE | 61% | 38% | 45% | 18% | 61% | 38% | | 20% |
| LLDPE | 61% | 38% | 45% | 18% | 61% | 38% | | 20% |
| HDPE | 61% | 38% | 45% | 18% | 61% | 38% | | 20% |
| PP | 61% | 38% | 45% | 8% | 61% | 38% | | 20% |
| PS | 61% | | 45% | 13% | 61% | | | 20% |
| PVC | 61% | 38% | 45% | 13% | 61% | 38% | | 20% |
| PET† | 61% | | | | | | | |
| PUR | | | | | | | | |
| Polyester fibre | | | | | | | | |
| Polyamide fibre | | | | | | | | |
| Other fibre | | | | | | | | |
| Rubber | | | | | | | | |
| Other thermoplastics | 61% | 38% | 45% | 13% | 61% | | | 20% |
| Other thermosets | | | | | | | | |

* set as the recycling rate as packaging materials.

# set as the recycling rate as vehicles in the transportation sector.

† note that 72% of recycled PET recyclates is used for polyester fibre and the remaining 28% is used for PET packaging materials[23].

$ textiles, such as polyester fibre and polyamide fibre, are not mechanically recycled at a large scale[21]. Recycled polyester fibre is mostly made of recycled PET bottles[24].

Figure S2 exemplifies the mass flow of LDPE used as packaging materials at the end-of-life. Since the mechanical recycling rates of polymers used in different sectors vary, this is only one example.

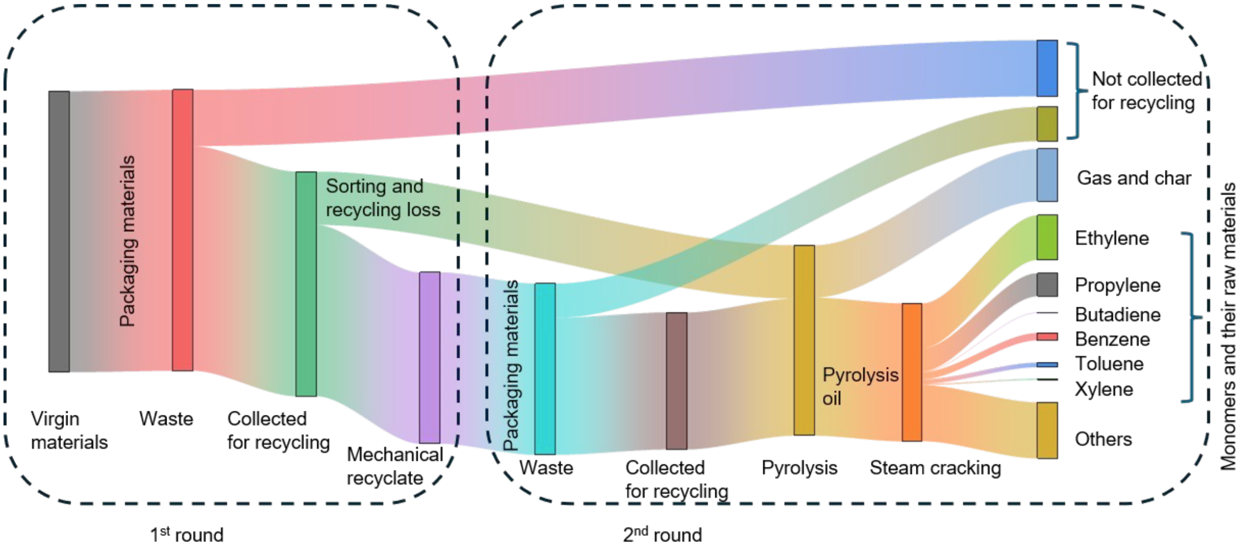


**Figure S2.** The mass flow of LDPE used as packaging materials in the chemical recycling with steam cracking scenario.

Figure S3 shows the historical and projected stock-in-service per capita in various sectors and countries. Norway has the highest stock per capita in all the sectors. The following sectoral stock per capita increased in the last decades and the slope dropped: transportation in Norway and Sweden, building and construction in Denmark, Iceland and Norway.

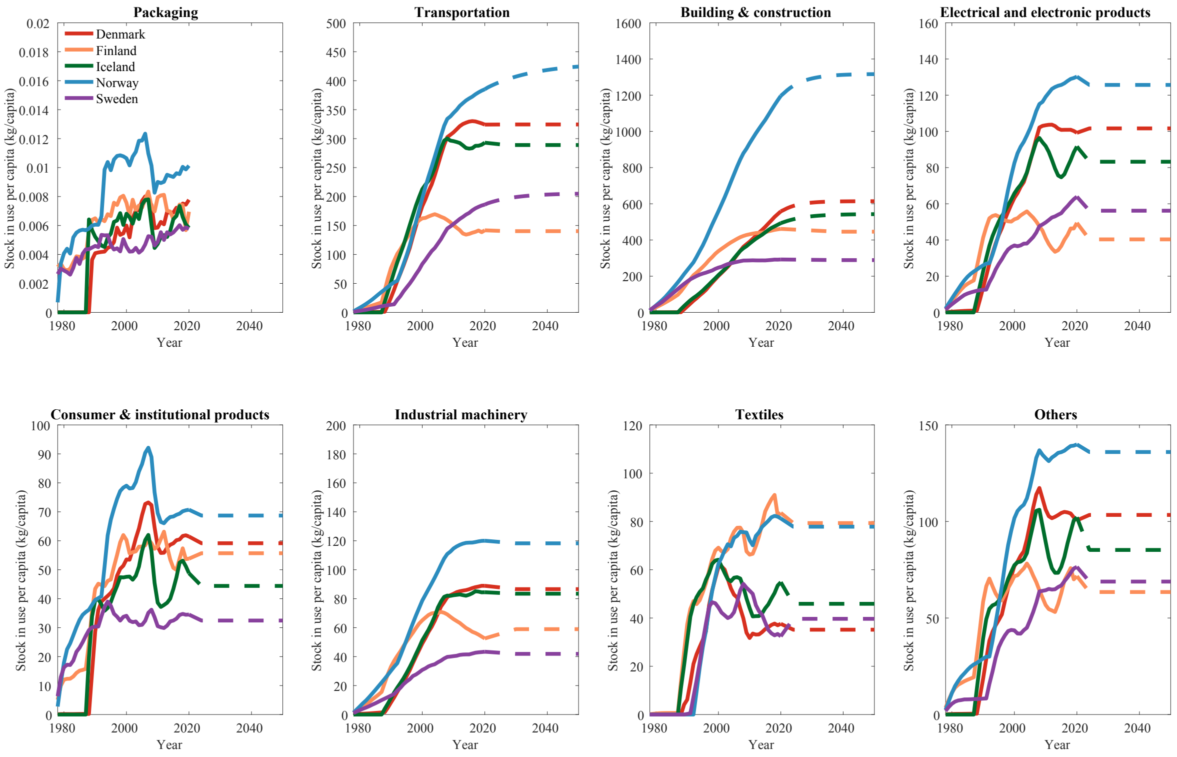


**Figure S3.** The national stock-in-service per capita in various sectors, except for packaging. The solid lines represent historical trends, while the dashed lines represent the projected stock-in-service per capita.

Figure S4 shows the historical and projected input flow of polymer packaging materials per capita in five Nordic countries.

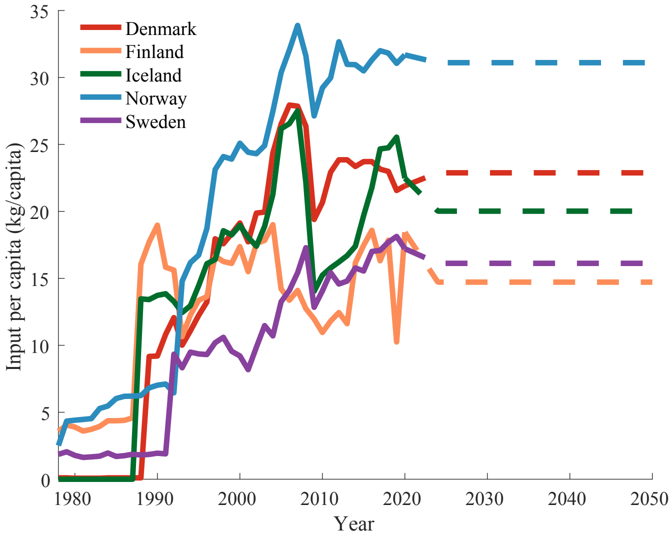


**Figure S4.** The national packaging input flow per capita. The solid lines represent historical trends, while the dashed lines represent the projected input flow per capita.

## 2. Model validation

The results published by the Danish Environmental Protection Agency are used to validate our model.[25] We used the mass flow in 2016 to be consistent with the report. Table S6 shows that most of the results are at the same order of magnitude, except for imported hidden polymers. The estimated 16,000 kt $a^{-1}$ in the report is two orders of magnitude higher than other imported streams, while our study used self-compiled list of commodities and fractions of polymers and had an estimated mass flow at the same order of magnitude of other imported products.

**Table S6.** Comparison of Danish mass flows in 2016 estimated by this study and the Danish Environmental Protection Agency[25]

| Variables | Reference[25] | This study |
|---|---|---|
| Imported primary polymers / kt $a^{-1}$ | 750 | 690 |
| Imported intermediate /semi-finished products / kt $a^{-1}$ | 240 | 270 |
| Imported final/finished products / kt $a^{-1}$ | 370 | 440 |
| Imported hidden polymers/plastic containing products / kt $a^{-1}$ | 16,000 | 380 |
| Exported primary polymers / kt $a^{-1}$ | 90 | 88 |
| Exported intermediate /semi-finished products / kt $a^{-1}$ | 140 | 190 |
| Exported final/finished products / kt $a^{-1}$ | 230 | 310 |
| Exported hidden polymers/plastic containing products / kt $a^{-1}$ | 16,000 | 310 |
| Recycled polymers / kt $a^{-1}$ | 58 | 43 |
| Domestic consumption / kt $a^{-1}$ | 1,000 | 980 |
| Imported plastic waste / kt $a^{-1}$ | 38 | 36 |
| Exported plastic waste / kt $a^{-1}$ | 59 | 89 |
| Incineration/ kt $a^{-1}$ | 420 | 700 |
| Other treatment/landfilling/ kt $a^{-1}$ | 11 | 28 |

## 3. **Results and discussions**

Figure S5 and S6 display the national historical and projected stock-in-service and input flow per sector, respectively. While building and construction sector has the highest stock-in-service because of the high average lifetime, packaging has the highest mass flow.

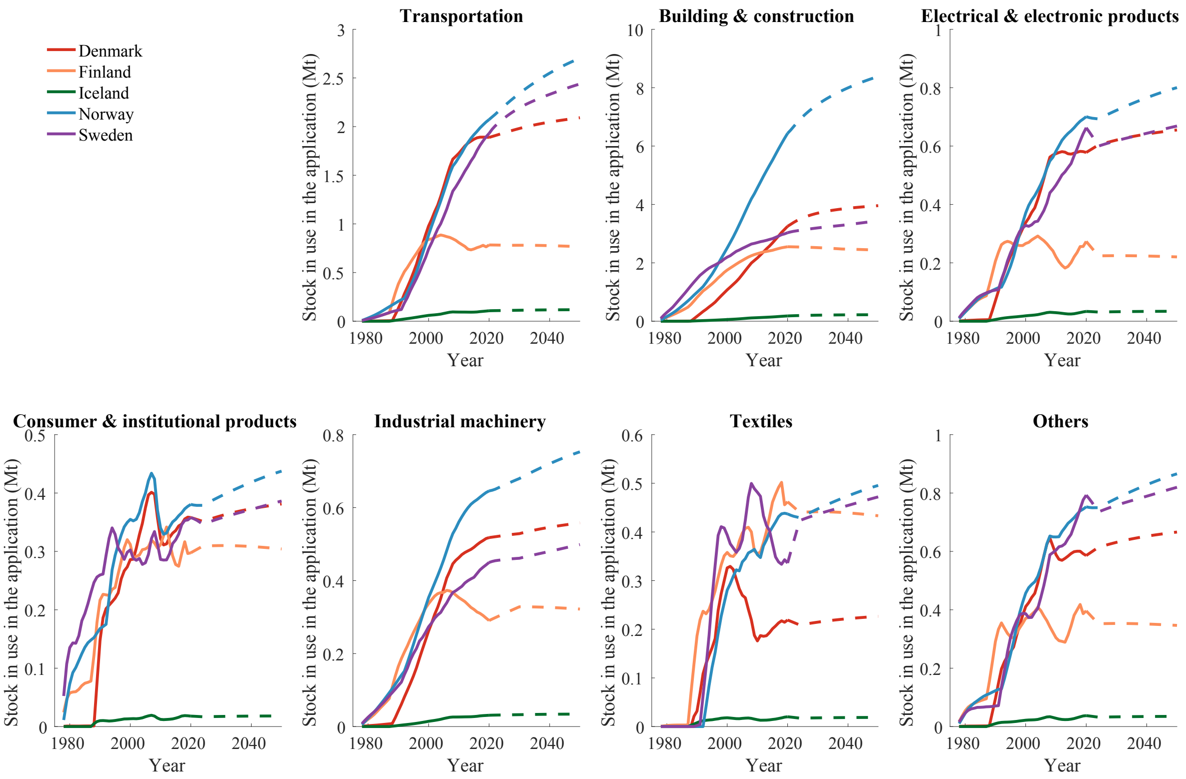


**Figure S5.** The historical and projected national stock in each application. The solid lines represent historical trends, while the dashed lines represent the projected input flow per capita.

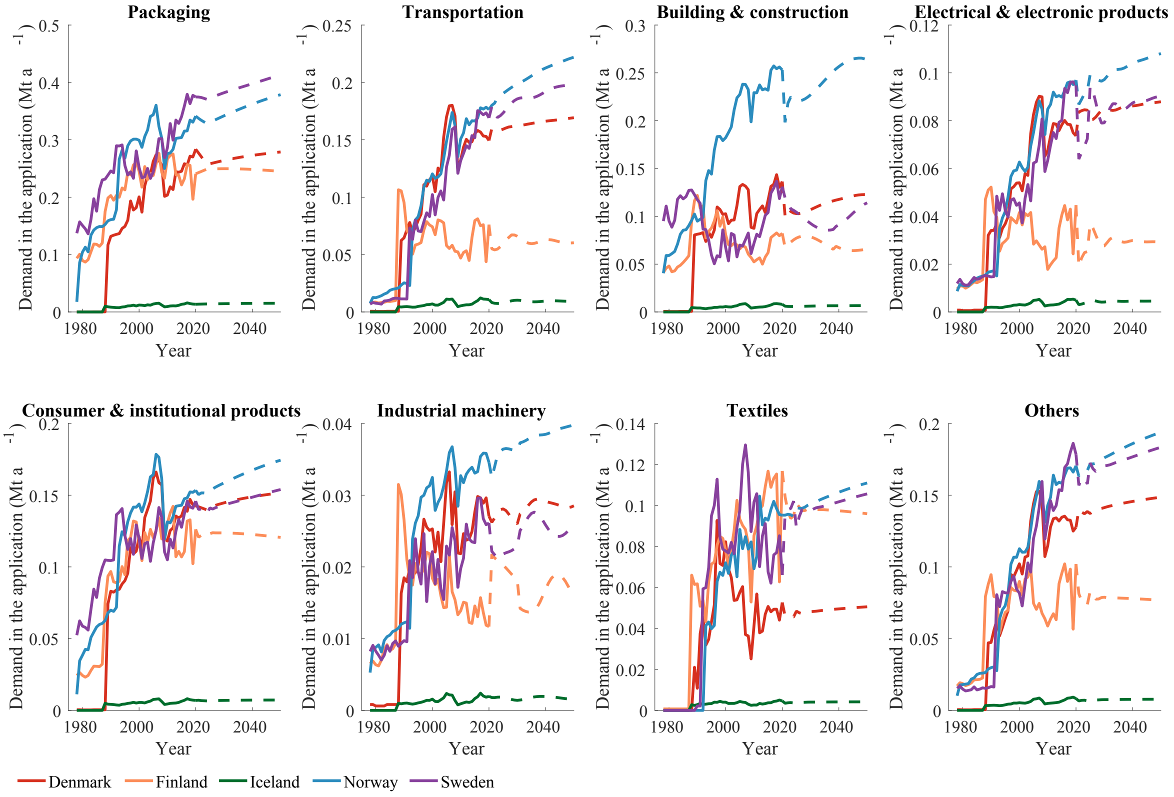


**Figure S6.** The historical and projected national input flow in each application. The solid lines represent historical trends, while the dashed lines represent the projected input flow per capita.

Figure S7 shows the national mass flow of polymer products and waste in Iceland, which is negligible compared with the other four Nordic countries due to the low population.

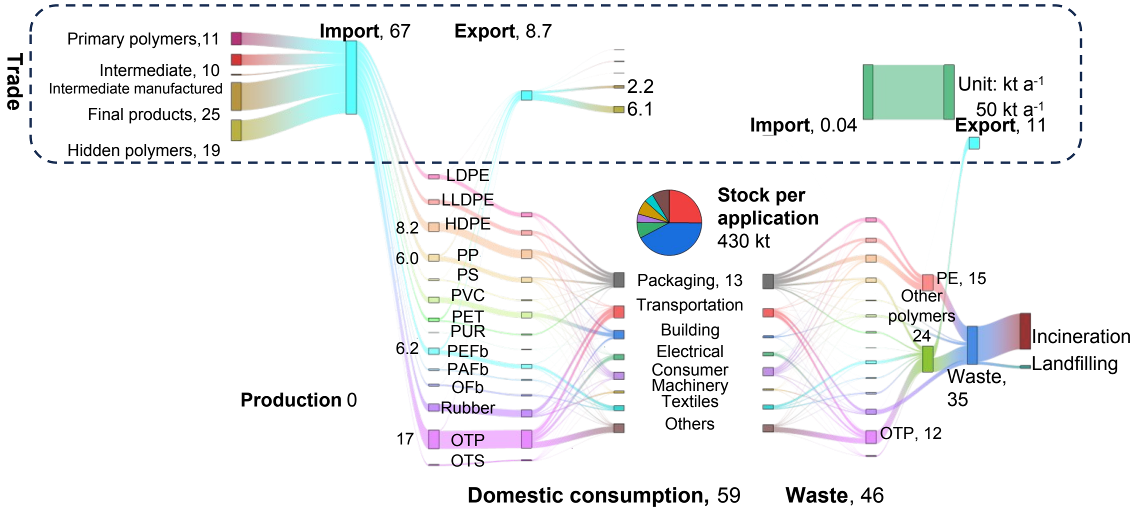


**Figure S7.** The national mass flow of polymers in 2020 in Iceland. The pie charts represent the amount of stock in-service in various applications. The thickness of the line stands for the mass flow of polymers in kilotons per annum (kt $a^{-1}$).

Figure S8 displays the allocation of input flow, stock-in-service and not-recycled waste to polymers, sectors and countries in the BAU scenario. Building and construction has the highest fraction of stock. Norway has the highest demand and waste generation.

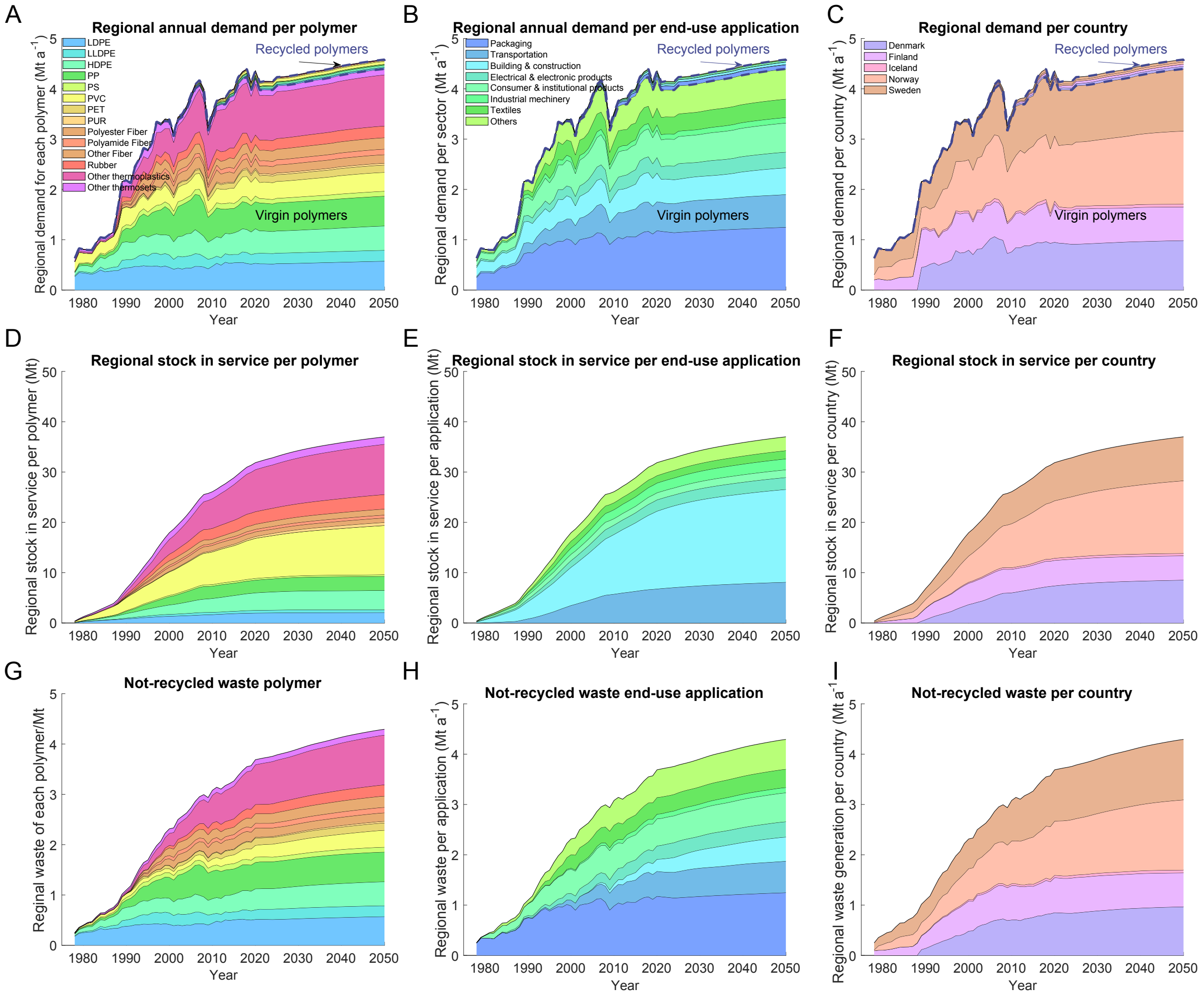


**Figure S8.** The projected regional demand, stock in service and not-recycled waste per polymer, end-use application and country in the BAU scenario.

Figure S9 shows the allocation of input flow, stock-in-service and not-recycled waste to polymers, sectors and countries in the enhanced mechanical recycling scenario. Recycling polymer wastes could not only reduce demand for virgin polymers, but also reduce waste to be processed by incineration, landfilling and mismanagement.

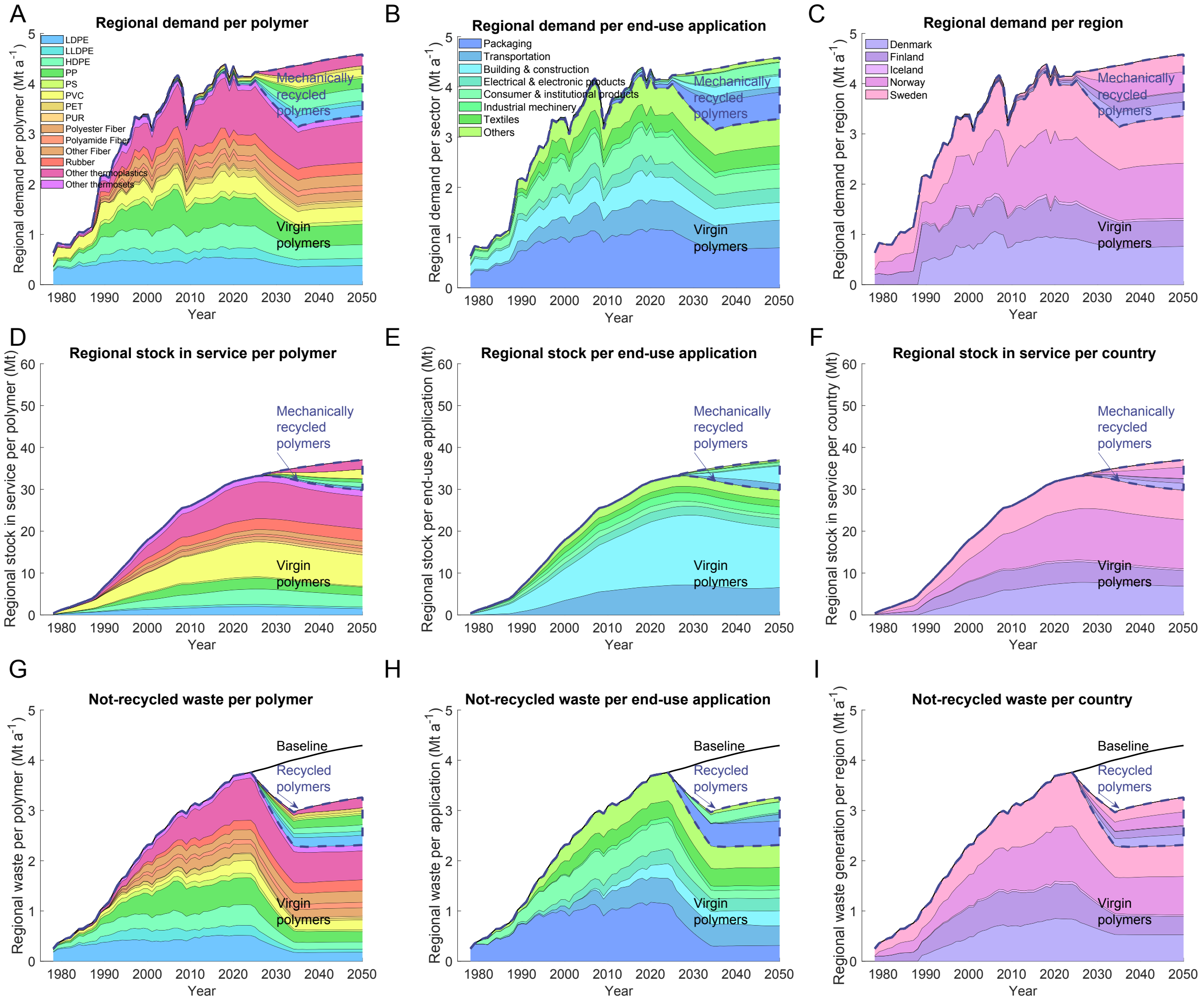


**Figure S9.** The projected regional demand, stock in service and not-recycled waste per polymer, end-use application and country in the enhanced mechanical recycling scenario.

Figure S10 shows the allocation of input flow, stock-in-service and not-recycled waste to polymers, sectors and countries in the chemical recycling without steam cracking scenario. The contribution of depolymerization and polymerization of suitable polymers could only contribute to 5% of the regional demand by 2050. The contribution of mechanical recycling is the same as the enhanced mechanical recycling.

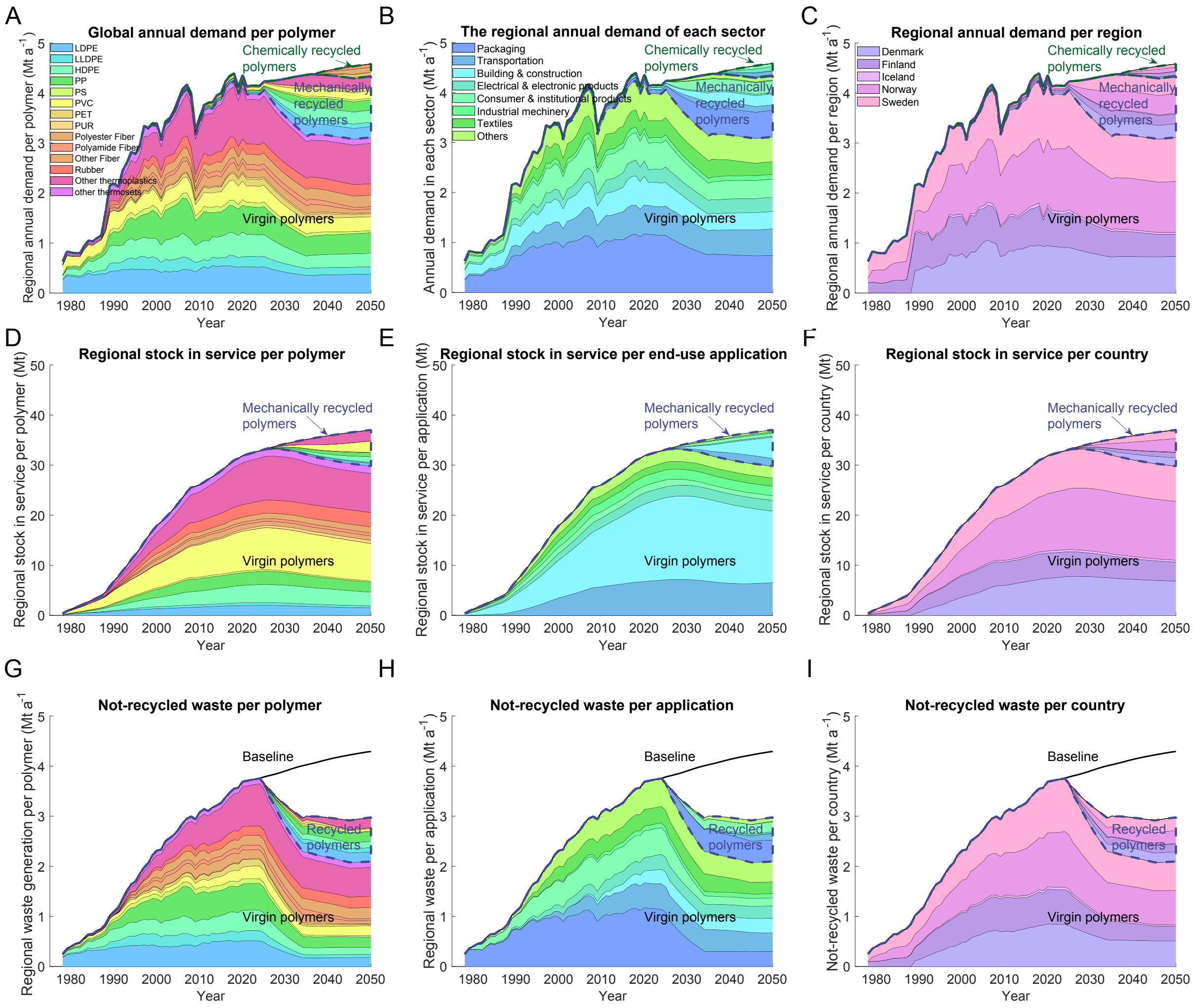


**Figure S10.** The projected regional demand, stock in service and not-recycled waste per polymer, end-use application and country in the chemical recycling without steam cracking scenario.

Figure S11 shows the allocation of input flow and not-recycled waste to polymers and countries in the chemical recycling with steam cracking scenario. Chemical recycling could potentially contribute to 22% of the regional demand in 2050. The amount of waste could be substantially reduced because of pyrolysis.

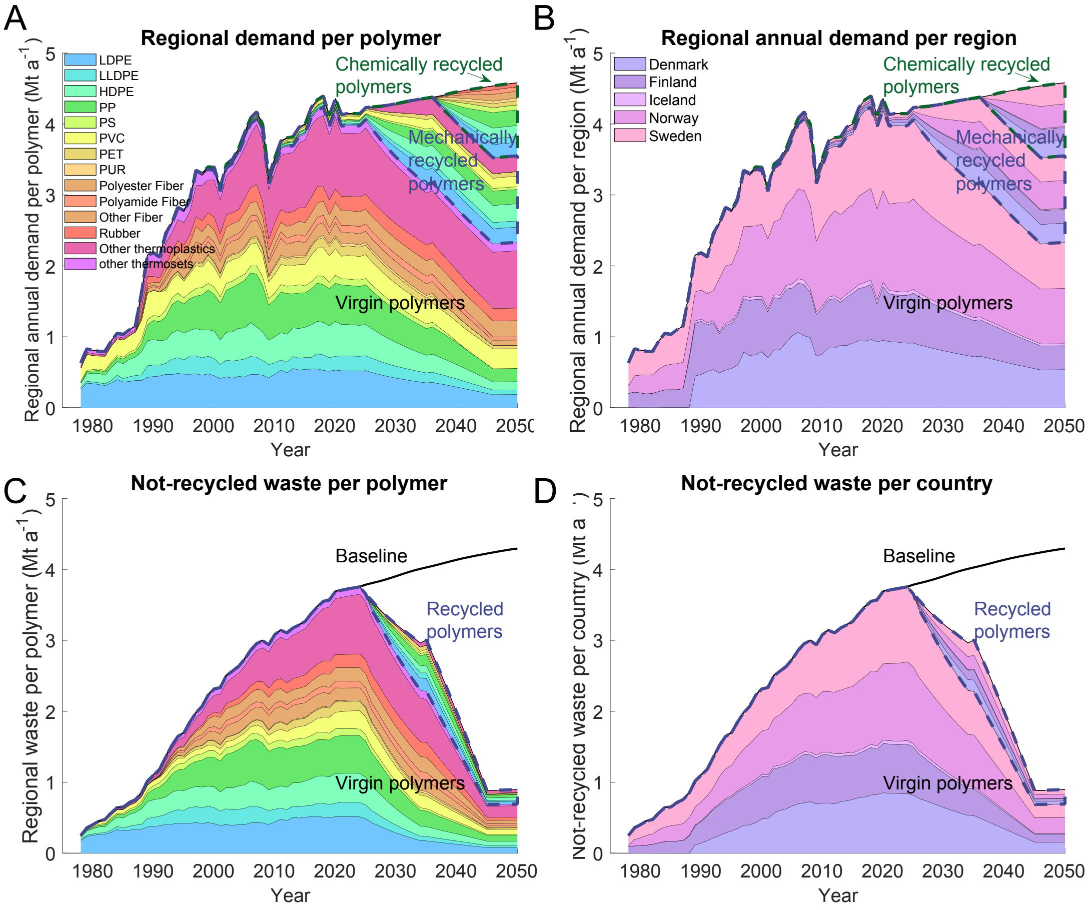


**Figure S11.** The projected regional demand and not-recycled waste per polymer and country in the chemical recycling with steam cracking scenario.

**Table S7.** Sensitivity analysis of virgin and recycled polymers in mechanical recycling scenario

| Polymers | | Standard mechanical recycling scenario | -20% recycling rate | | +20% recycling rate | |
|---|---|---|---|---|---|---|
| | | | Result | Change in result | Result | Change in result |
| Virgin polymers | LDPE | 0.38 | 0.41 | 8.1% | 0.35 | -7.1% |
| | LLDPE | 0.15 | 0.16 | 8.3% | 0.13 | -7.2% |
| | HDPE | 0.27 | 0.31 | 14.4% | 0.24 | -12.7% |
| | PP | 0.41 | 0.44 | 7.0% | 0.38 | -6.0% |
| | PS | 0.07 | 0.07 | 7.4% | 0.07 | -6.4% |
| | PVC | 0.28 | 0.31 | 9.8% | 0.25 | -9.6% |
| | PET | 0.11 | 0.11 | 8.2% | 0.10 | -7.0% |
| | PUR | 0.04 | 0.04 | 0.0% | 0.04 | 0.0% |
| | polyester fiber | 0.17 | 0.17 | 0.0% | 0.17 | 0.0% |
| | polyamide fiber | 0.11 | 0.11 | 0.0% | 0.11 | 0.0% |
| | other fiber | 0.23 | 0.23 | 0.0% | 0.23 | 0.0% |
| | Rubber | 0.23 | 0.23 | 0.0% | 0.23 | 0.0% |
| | Other thermoplastics | 0.81 | 0.84 | 4.3% | 0.78 | -3.8% |
| | Other thermosets | 0.12 | 0.12 | 0.0% | 0.12 | 0.0% |
| Recycled polymers | LDPE | 0.22 | 0.19 | -14.2% | 0.24 | 12.5% |
| | LLDPE | 0.09 | 0.08 | -13.8% | 0.10 | 12.1% |
| | HDPE | 0.24 | 0.20 | -16.2% | 0.28 | 14.3% |
| | PP | 0.20 | 0.17 | -14.0% | 0.23 | 12.1% |
| | PS | 0.04 | 0.03 | -14.0% | 0.04 | 12.2% |
| | PVC | 0.15 | 0.12 | -18.5% | 0.18 | 18.0% |
| | PET | 0.06 | 0.06 | -13.5% | 0.07 | 11.5% |
| | PUR | 0.00 | 0.00 | | 0.00 | |
| | polyester fiber | 0.00 | 0.00 | | 0.00 | |
| | polyamide fiber | 0.00 | 0.00 | | 0.00 | |
| | other fiber | 0.00 | 0.00 | | 0.00 | |
| | Rubber | 0.00 | 0.00 | | 0.00 | |
| | Other thermoplastics | 0.22 | 0.19 | -15.8% | 0.25 | 14.0% |
| | Other thermosets | 0.00 | 0.00 | | 0.00 | |

## References


(1) Danish Environmental Protection Agency. *Preliminary Assessment of Plastic Material Flows in Denmark: Technical Report*, Environment Project no. 2090.; 2019.

(2) Ministry of Environment and Food. *Plastics without Waste – The Danish Government's Plastics Action Plan*; 2018. https://en.fvm.dk/Media/638499759917701981/Regeringens_plastikhandlingsplan_UK.pdf.

(3) McKinsey & Company; Innovation Fund Denmark. *New Plastics Economy: A Research Innovation and Business Opportunity for Denmark*; 2019; p 69. https://innovationsfonden.dk/sites/default/files/2019-01/new-plastics-report-jan16-2019-vf.pdf.

(4) Eurostat. *Plastic packaging waste: 38% recycled in 2020*. https://ec.europa.eu/eurostat/web/products-eurostat-news/-/ddn-20221020-1.

(5) Tilastokeskus. *Waste treatment by type of treatment, 2017-2021*. PxWeb. https://pxdata.stat.fi:443/PxWebPxWeb/pxweb/en/StatFin/StatFin__jate/statfin_jate_pxt_12qy.px/.

(6) Veera, N. *Finland has responded to the EU's early warning concerning the recycling rate of plastic packaging*. Verkkolehti - Luotettavaa tietoa pakkauskierrätyksestä ja tuottajavastuusta. https://verkkolehti.rinkiin.fi/finland-has-responded-to-the-eus-early-warning-concerning-the-recycling-rate-of-plastic-packaging?lang=en.

(7) Judl, J.; Horn, S.; Karppinen, T. K. M. Towards a Low-Carbon Plastic Waste Recycling in Finland: Evaluating the Impacts of Improvement Measures on GHG Emissions. *Circ. Econ. Sustain.* **2024**, *4* (1), 755–776. https://doi.org/10.1007/s43615-023-00306-w.

(8) RINKI. *Packaging statistics*. Rinkiin.fi. https://rinkiin.fi/en/about-rinki/packaging-statistics/.

(9) Abbasi, G.; Hauser, M.; Baldé, C. P.; Bouman, E. A. A High-Resolution Dynamic Probabilistic Material Flow Analysis of Seven Plastic Polymers; A Case Study of Norway. *Environ. Int.* **2023**, *172*, 107693. https://doi.org/10.1016/j.envint.2022.107693.

(10) EastCham Finland. *Textile*. https://www.eastcham.fi/finnishwastemanagement/municipal-solid-waste/recycling-and-recovery/textile/.

(11) Lena, S.; Hanna, L. N.; Anna-Karin, W. *Plastic in Sweden Facts and Practical Advice. A Short Version of Kartläggning Av Plastflöden i Sverige (Mapping Plastic Flows in Sweden)*; Naturvårdsverket, 2019. https://www.naturvardsverket.se/globalassets/media/publikationer-pdf/8800/978-91-620-8854-5.pdf.

(12) European Environment Agency. *Early Warning Assessment Related to the 2025 Targets for Municipal Waste and Packaging Waste (Sweden)*; 2022; p 55. https://www.eea.europa.eu/publications/many-eu-member-states/sweden/view.

(13) Anna, F.; Sara, A.; Cecilia, A.; Nils, B.; Maja, D.; Jurate, M.-P.; Hanna, U.; Martin, V. och M. G. *Kartläggning av plastflöden i Sverige 2020*; Rapport 7038; 2022; p 229. https://www.naturvardsverket.se/publikationer/7000/978-91-620-7038-0/.

(14) Milios, L.; Esmailzadeh Davani, A.; Yu, Y. Sustainability Impact Assessment of Increased Plastic Recycling and Future Pathways of Plastic Waste Management in Sweden. *Recycling* **2018**, *3* (3), 33. https://doi.org/10.3390/recycling3030033.

(15) bishop. *Swedish Plastic Recycling - We give new life to plastic*. Svensk Plaståtervinning. https://www.svenskplastatervinning.se/en/.

(16) Anderson, S. *Plastic in Sweden - Facts and Practical Advice: Data from 2020*; Swedish Environmental Protection Agency (Naturvårdsverket): Stockholm, 2022.

(17) Grip, L. Mapping Flows of Textile Waste in Sweden and an Analysis of Alternatives. *Lunds Univ.* **2018**.
(18) Yunhu Gao; Cabrera Serrenho, A. Evaluating the Potential to Reduce the Global Demand for Virgin Polymers. *Rev.*
(19) SYSTEMIQ. *ReShaping Plastics: Pathways to a Circular, Climate Neutral Plastics System in Europe.*; 2022.
(20) Eriksen, M. K.; Pivnenko, K.; Faraca, G.; Boldrin, A.; Astrup, T. F. Dynamic Material Flow Analysis of PET, PE, and PP Flows in Europe: Evaluation of the Potential for Circular Economy. *Environ. Sci. Technol.* **2020**, *54* (24), 16166–16175. https://doi.org/10.1021/acs.est.0c03435.
(21) Klotz, M.; Haupt, M.; Hellweg, S. Potentials and Limits of Mechanical Plastic Recycling. *J. Ind. Ecol.* **2023**, *27* (4), 1043–1059. https://doi.org/10.1111/jiec.13393.
(22) Meng, F.; Wagner, A.; Kremer, A. B.; Kanazawa, D.; Leung, J. J.; Goult, P.; Guan, M.; Herrmann, S.; Speelman, E.; Sauter, P.; Lingeswaran, S.; Stuchtey, M. M.; Hansen, K.; Masanet, E.; Serrenho, A. C.; Ishii, N.; Kikuchi, Y.; Cullen, J. M. Planet-Compatible Pathways for Transitioning the Chemical Industry. *Proc. Natl. Acad. Sci.* **2023**, *120* (8), e2218294120. https://doi.org/10.1073/pnas.2218294120.
(23) Shen, L.; Worrell, E.; Patel, M. K. Open-Loop Recycling: A LCA Case Study of PET Bottle-to-Fibre Recycling. *Resour. Conserv. Recycl.* **2010**, *55* (1), 34–52. https://doi.org/10.1016/j.resconrec.2010.06.014.
(24) Textile Exchange. *Preferred Fiber & Materials Market Report 2020*; 2022.
(25) Pivnenko, K.; Damgaard, A.; Astrup, T. F. *Preliminary Assessment of Plastic Material Flows in Denmark: Technical Report*; Danish Environmental Protection Agency, 2019.
